# Arteriovenous Malformation of the Oral Cavity

**DOI:** 10.1155/2014/353580

**Published:** 2014-02-10

**Authors:** S. M. Manjunath, Sujan Shetty, Ninad J. Moon, Bhushan Sharma, Kiran Kumar Metta, Nitin Gupta, Sandeep Goyal, Simranjit Singh

**Affiliations:** ^1^Department of Oral Pathology & Microbiology, MM College of Dental Sciences, MM University, Mullana, Ambala, Haryana 133207, India; ^2^Department of Periodontics, RKDF Dental College and Research Centre, Bhopal, Madhya Pradesh 462026, Madhya Pradesh, India; ^3^Department of Oral Pathology & Microbiology, Gian Sagar Dental College, Rajpura, Punjab 140401, India; ^4^Department of Conservative Dentistry and Endodontics, MIDSR Dental College and Hospital, Latur, Maharashtra 413531, India; ^5^Baba Jaswant Singh Dental College, Ludhiana, Punjab 141010, India; ^6^Department of Oral Pathology & Microbiology, Surendera Dental College and Research Institute, Sriganganagar, Rajasthan 335001, India; ^7^Department of Oral Pathology & Microbiology, Dr. Harvansh Singh Judge Institute of Dental Sciences and Hospital, Panjab University, Chandigarh 160014, India

## Abstract

Vascular anomalies are a heterogeneous group of congenital blood vessel disorders more typically referred to as birthmarks. Subcategorized into vascular tumors and malformations, each anomaly is characterized by specific morphology, pathophysiology, clinical behavior, and management approach. Hemangiomas are the most common vascular tumors. Lymphatic, capillary, venous, and arteriovenous malformations make up the majority of vascular malformations. Arteriovenous malformation of the head and neck is a rare vascular anomaly but when present is persistent and progressive in nature and can represent a lethal benign disease. Here we present a case report of a 25-year-old male patient with arteriovenous malformation involving the base of tongue.

## 1. Introduction

“Vascular malformation” is a generalized term used to describe a group of lesions, present at birth, formed by an anomaly of angiovascular or lymphovascular structures. Vascular malformations occur in approximately 1% of births but majority of these patients do not present for treatment [1]. The high-flow vascular anomalies in the head and neck are arteriovenous malformations (AVMs) [2]. These are the lesions with direct communications between an artery (or arteries) and a vein (or veins) bypassing the capillary bed [3].

AVMs are usually present at birth but commonly manifest in childhood or adolescence. These lesions can occur at any area of the body [1]. They have gradual onset and progression [3]. In the oral cavity, these can present at any site, but most commonly occur on anterior two-thirds of the tongue, palate, and gingival and buccal mucosa [4].

These lesions can be diagnosed by plain radiography, computed tomography scans, magnetic resonance imaging, or angiography. Various sclerosing agents and embolization, combined with surgical treatment,are still the most conventional modern approach to treat these lesions [5]. In this paper, we present a case of arteriovenous malformation involving the base of tongue of a 25-year-old male followed by a review of the literature.

## 2. Case Report

A 25-year-old male patient with a noncontributory medical history reported to the Department of Oral and Maxillofacial Pathology with the chief complaint of multiple swellings on left side of face and inside the mouth. The swellings were not associated with any pain and had gradually increased to the present status. A similar swelling was revealed on the back of his father.

**Figure 1:**
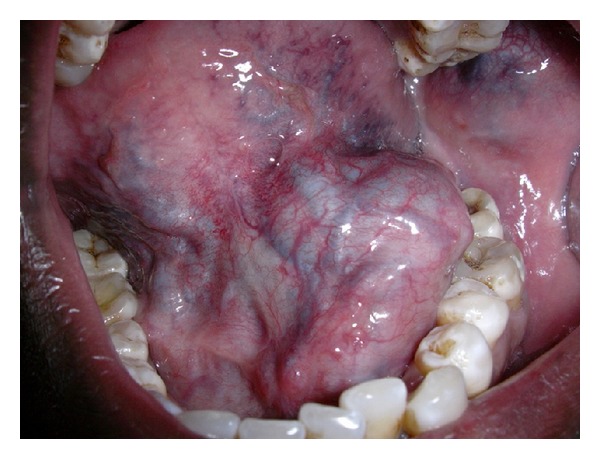
Intraoral photograph showing swelling with bluish discoloration in floor of the mouth with intact overlying mucosa.

Extraoral examination revealed no significant facial asymmetry. The swelling was poorly defined and had increased in size in a dependent position. On palpation, it was soft in consistency and was easily compressible but lacked prominent pulsation. Intraoral examination revealed swelling with bluish discoloration in floor of the mouth with intact overlying mucosa (Figure 1). The swelling was soft in consistency and nontender on palpation, readily blanched with compression, and lacked a prominent pulsation. The swelling extended from the left mandibular central incisor anteriorly to the third molar region posteriorly. Bluish discoloration was also seen in the lower labial vestibule in relation to left lateral incisor and left canine and in the left buccal mucosa with respect to left first and second maxillary molars.

**Figure 2:**
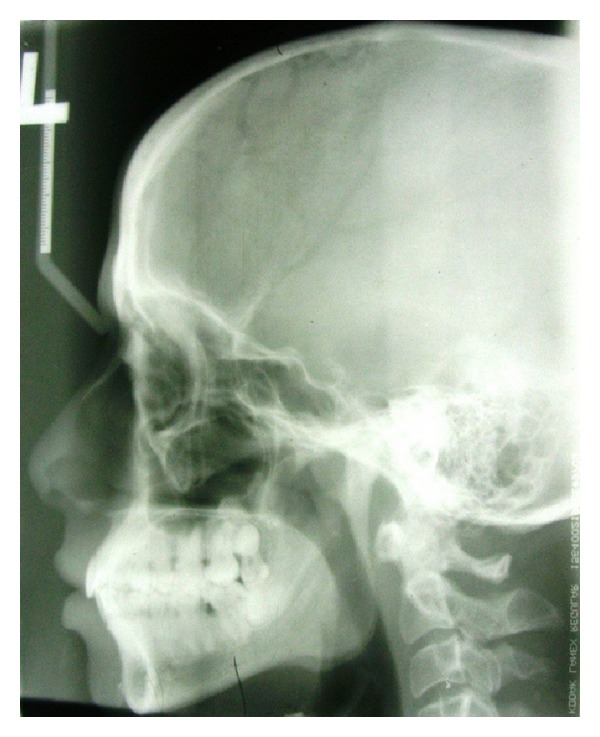
Plain radiograph revealing no bony involvement, but phleboliths can be noticed.

**Figure 3:**
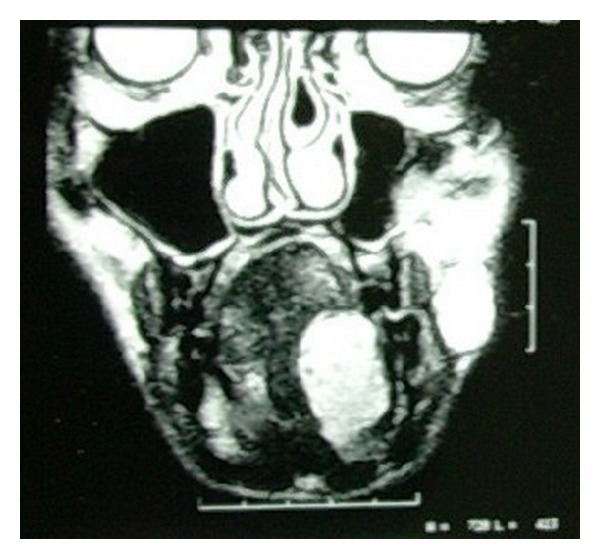
MRI showing hyperintense T2-weighted images with no flow voids.

**Figure 4:**
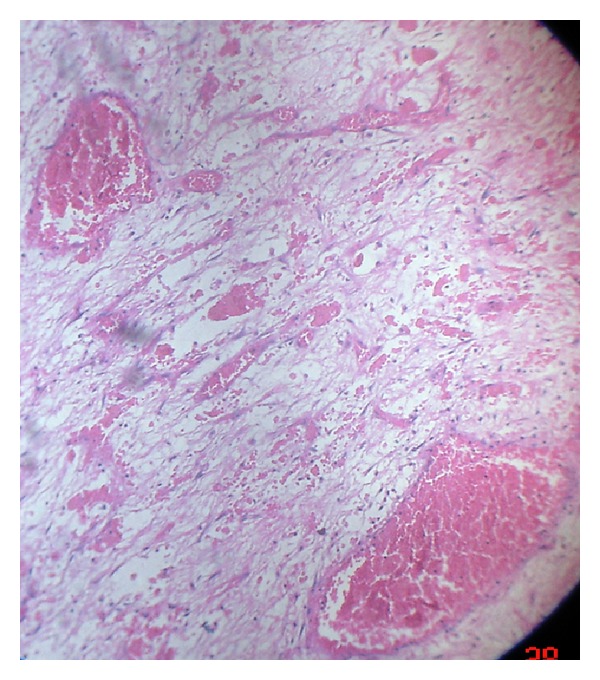
Photomicrograph showing the presence of large cavernous spaces lined by endothelial cells [10X].

Blood was examined for routine investigations and all the values were found to be normal. Plain radiographs revealed no bony involvement, but phleboliths were noticed (Figure 2). MRI revealed hyperintense T2-weighted images with no flow voids (Figure 3). Biopsy was performed and on histopathological examination it revealed the presence of large cavernous spaces lined by endothelial cells. These spaces were filled with blood and supported by fibrous connective tissue stroma (Figure 4).

MRI impression and histopathological findings revealed the diagnosis of arteriovenous malformation involving base of tongue.

## 3. Discussion

Identification and classification of vascular anomalies were hampered historically by the use of confusing nomenclature. Early classifications published by Virchow and Wagner characterized vascular lesions according to the vessel's pathologic appearance. Vascular growths were divided into angiomas and lymphangiomas. The biologic behavior and natural history of the vascular lesions were not considered. In 1982, Mulliken and Glowacki made great strides to dispel this confusion when they published a classification of vascular birthmarks, grouping them into two major categories: hemangiomas and malformations. More specifically, hemangiomas were differentiated from vascular malformations by their clinical appearance, histopathologic features, and biologic behavior. In 1996, the classification was modified slightly to reflect the importance of other types of vascular tumors that exhibit different clinical and histologic characteristics than the common infantile hemangioma, including kaposiform hemangioendotheliomas, tufted angiomas, and others. Consequently, the updated International Society for the Study of Vascular Anomalies/biologic classification divides vascular birthmarks into vascular tumors and vascular malformations [7].

A vascular malformation can be slow-flow (i.e., capillary, lymphatic, or venous) or fast-flow (i.e., arterial). If there are combinations of these elements, the malformation is called an arteriovenous malformation (AVM), lymphatico-venous malformation (LVM), or capillary-lymphatico-venous malformation (CLVM) [8]. Arteriovenous malformations are high-flow lesions with direct communications between an artery (or arteries) and a vein (or veins), bypassing the capillary bed [3]. The earliest description of AVM was the reporting of snakes covering the Greek God Gordon's head.” [9]. Arteriovenous malformation of the head and neck is a rare vascular anomaly but when present is persistent and progressive in nature and can represent a lethal benign disease [1].

They can be classified as

1. congenital: occuring as a result of lack of differentiation of arteries, capillaries, and veins during vascular development,
2. acquired: associated with previous history of injury/trauma.

Little is known about the origin and pathogenesis of AVM. Defects in TGF-beta signaling and a genetic two-hit hypothesis are the prevailing theories to the pathogenesis. Trauma, ischaemic event secondary to thrombosis, ectasia, hormonal changes, and puberty can induce proliferation of the AVM and trigger the growth of the lesion and manifestation of its troublesome symptoms [10]. These are often the cause of massive, sometimes fatal, hemorrhages [5]. Trauma, ischaemic event secondary to thrombosis, ectasia, and hormonal changes all serve as risk factors. Puberty can also induce proliferation of the AVM and trigger the growth of the lesion and manifestation of its troublesome symptoms [6]. Progesterone receptors have been isolated in AVMs explaining their expansion during puberty [11].

AVMs are usually present at birth but commonly manifest in childhood or adolescence. Our case was associated with a 25-year-old male. AVM has a gradual onset and progression; it is rarely associated with an enlarged heart and high output cardiac failure. In the present case also; the patient did not reveal any history of heart failure. There are a series of cases described by different authors but one of the largest series was reported by Kohout et al. who reported on 81 AVMs located in the head and neck areathe majority ofwhich were localized over the cheek (31%), ear (16%), nose (10%), forehead (10%), upper lip (7%), mandible (5%), neck (5%), scalp (4%), and maxilla (4%) [12].

These lesions present as a pulsatile mass with a thrill, bruit, and occasionally local hyperthermia, ulceration or bleeding, functional impairment due to arterialsteal, and ischaemia [3]. Shunting of blood diminishes nutritive flow, which may result in skin necrosis, ulceration, and bleeding [2]. Many lesions have either a warm erythematous blush or a true port-wine stain in the overlying skin [2].

In the oral cavity, these can present at any site, but most commonly on anterior two-thirds of tongue, leading to macroglossia and difficulty in mastication, speech, and deglutition. Other sites that may be involved are palate, gingiva, and buccal mucosa [4]. Intraosseous VMs near the alveolar bone are often present with pericoronal bleeding, mobile teeth, and sometimes occlusal anomalies [13]. The present case showed the involvement of multiple sites, that is floor of mouth, bucal mucosa, and lower labial vestibule. AVMs are by far the most difficult vascular anomaly to manage due to the replacement of normal tissue by disease vessels and very high recurrence rates. Vascular naevi or phlebectasias may discolour the adjacent mucosa or skin. In the present case the lesions were associated with bluish discoloration.

Vascular malformations may be associated with underlying disease or systemic anomalies in select situations. The syndromes known to be associated with arteriovenous malformations include Bonnet-Dechaume-Blanc syndrome or Wyburn-Mason syndrome, Parkes-Weber syndrome, Capillary malformation-AVM syndrome, and Cobb syndrome [14].

Vascular tumors and anomalies exhibit a range of coagulopathies. Extensive vascular malformations are known to be associated with a form of consumption coagulopathy. A chronic coagulopathy could have serious implications for a patient's ability to respond to sclerotherapy or embolization. Severe deficiency of normal clotting factors or platelets may prevent a successful response to treatment. Occasionally, to facilitate successful thrombosis, cryoprecipitate, platelets, or fresh frozen plasma has to be administered to patients with chronic coagulopathy (such as low platelets, low fibrinogen, or positive d-dimers) before performing sclerotherapy or embolization. Furthermore, kaposiform hemangioendothelioma and tufted angioma may manifest thrombocytopenia and coagulopathy known as kasabach-Merritt syndrome. Vascular anomalies, including Klippel-Trenaunay and Parkes-Weber syndromes, have been reported to manifest both chronic consumption coagulopathies and acute changes in response to surgical procedures, such as fracture reduction or surgery related to childbirth [15].

Plain radiography and computed tomography scans have a limited role as diagnostic tools in high-flow vascular malformations. Radiographically, in the mandible and maxilla, the lesion produces a poorly defined, radiolucent image, often having the appearance of a honeycomb or soap bubbles, with small rounded and irregular lacunae. Magnetic resonance imaging (MRI) has become the investigation of choice since it depicts the extent and lack of invasion of these lesions. Angiography is useful in poorly defined cases and for embolization before surgery. It demonstrates the flow characteristics, feeding vessels, and dangerous anastomoses.

AVMs present a therapeutic challenge because of their haemodynamic characteristics and their modality of growth. Surgical resection is often associated with extensive blood loss and an incomplete resection frequently leads to regrowth of the tumour to sizes that are often larger than its original size. Proximal ligation of the parent vessel should be avoided as it is ineffective and may aggravate the problem making future endovascular therapy difficult or impossible [16].

If an AVM is asymptomatic, no treatment is necessary. But with complications, such as pain, ulceration, bleeding, or heart failure, therapy is necessary [5]. Multimodal treatment, including preoperative embolization and complete surgical resection, is usually necessary for the management of AVMs [7]. Various sclerosing agents (sodium morrhuate, boiling water, nitrogen mustard, etc.) have been used to treat these high-flow lesions but have proven ineffective because they were displaced from their site of action by the speed of the blood flow [5]. This problem can be overcome by using preoperative embolization of the feeding arteries which reduces the hypervascularity and therefore aids in surgical resection of these lesions. At present, the embolic materials generally employed are absorbable gelatine foam, polyvinyl alcohol (PVA), absolute alcohol, and NBCA. Complications from embolization are infrequently seen; however, necrosis of adjacent tissues may occur. Hence, patients must be adequately treated with broad-spectrum antibiotics [16].

Highly selective embolization as a single treatment modality is rarely successful with high-flow lesions because of the later development of new vascular pathways. However, it leads to a significant reduction in the blood flow within the vascular tumour which decreases operative blood loss and permits complete resection of the tumour [16].

Recently, the direct transosseous puncture of the vascular bed has been proposed. Lesional curettage without resection preserves good bone support, but the excision is often deemed inadequate. Embolization, combined with surgical treatment, is still the most conventional modern approach [5]. The present case, considering the size of the lesion, was also treated using the combined approach of embolization and surgery.

## 4. Conclusion

A rare case of arteriovenous malformation of base of tongue in a 25-year-old male patient has been presented. The rareness of AVMs is equaled only by the morbidity they cause and the urgency of the measures to be taken once detected, in all circumstances. A high degree of suspicion leads to their diagnosis and considerably reduces the risks of a catastrophe once identified [5].
